# Dual sensor measurement shows that temperature outperforms pH as an early sign of aerobic deterioration in maize silage

**DOI:** 10.1038/s41598-021-88082-1

**Published:** 2021-04-22

**Authors:** Guilin Shan, Wolfgang Buescher, Christian Maack, André Lipski, Ismail-Hakki Acir, Manfred Trimborn, Fabian Kuellmer, Ye Wang, David A. Grantz, Yurui Sun

**Affiliations:** 1 Department of Agricultural Engineering, The University of Bonn, 53115 Bonn, Germany; 2 Institute of Nutrition and Food Science, The University of Bonn, 53115 Bonn, Germany; 3 Department of Botany and Plant Sciences, Kearney Agricultural Center, University of California at Riverside, Parlier, CA 93648 USA

**Keywords:** Chemical engineering, Metabolomics, Applied microbiology

## Abstract

High quality silage containing abundant lactic acid is a critical component of ruminant diets in many parts of the world. Silage deterioration, a result of aerobic metabolism (including utilization of lactic acid) during storage and feed-out, reduces the nutritional quality of the silage, and its acceptance by animals. In this study, we introduce a novel non-disruptive dual-sensor method that provides near real-time information on silage aerobic stability, and demonstrates for the first time that in situ silage temperature (T_si_) and pH are both associated with preservation of lactic acid. Aerobic deterioration was evaluated using two sources of maize silage, one treated with a biological additive, at incubation temperatures of 23 and 33 °C. Results showed a time delay between the rise of T_si_ and that of pH following aerobic exposure at both incubation temperatures. A 11 to 25% loss of lactic acid occurred when T_si_ reached 2 °C above ambient. In contrast, by the time the silage pH had exceeded its initial value by 0.5 units, over 60% of the lactic acid had been metabolized. Although pH is often used as a primary indicator of aerobic deterioration of maize silage, it is clear that T_si_ was a more sensitive early indicator. However, the extent of the pH increase was an effective indicator of advanced spoilage and loss of lactic acid due to aerobic metabolism for maize silage.

## Introduction

Aerobic deterioration of silage is unavoidable, particularly during feed-out^1–3^ because, once a silo is opened, air freely accesses the exposed silo face. Undesirable microorganisms, mainly yeast and acid-tolerant bacteria, proliferate under these newly aerobic conditions, metabolizing residual sugars, lactic and other organic acids to CO_2_, H_2_O and heat^4,5^. Consequently, silage temperature (T_si_) increases and the silage mass becomes aerobically unstable. To evaluate this process with a quantitative threshold, the aerobic stability of silage has variously been defined as the time after opening for T_si_ to reach 1.7 °C^6^ above ambient temperature, 2 °C^7–10^, 2.5 °C^11^ or 3 °C^2,12,13^, in order to evaluate effects of silage additives^7–9,13^, assess roles of microorganismse^2,12^, determine spatial patterns of T_si_^10^, assess impacts of site-specific bulk density in silage masses^6^, as well as various environmental effects^10^.

Since utilization of acids by aerobic microorganisms leads to a rise of silage pH, the pH of a silage mass reflects the extent of aerobic deterioration^14^. Previous studies of aerobic responses of silage pH used laboratory mini-silos^7,15–18^ and farm silos^5,10,19–23^, both with invasive sub-sampling followed by liquid extraction of silage samples. Such ex situ pH determination may interfere with silage conditions, including the anaerobic environment, and is typically completed at daily (or longer) time intervals, which obscures important short term dynamic changes. A recent on-farm study using several silos suggested that pH may be a less sensitive indicator of spoilage progression in maize silage than T_si_^21^.

A recent review^24^ suggested that an early indicator of aerobically unstable silage likely to substantially spoil within 24 h is when lactate-assimilating yeast counts are > 10^5^ cfu/g silage and, once they reach ~ 10^7^ cfu/g, silage pH and temperature begin to increase^24,25^. Since samples sent to a forage laboratory for plate-culture counts create assessment delays of several days, an early indicator of aerobic deterioration that can be instantly measured in situ is needed to allow decisions to be made on commercial silage masses in real time.

The objectives of the study were to (1) introduce a novel method using an advanced pH sensor combined with a temperature sensor for continuous and simultaneous measurements of maize silage; (2) characterize relationships between pH and T_si_ determined in situ following aerobic exposure of maize silage; and (3) evaluate aerobic loss of lactic acid associated with the temporal courses of T_si_ and pH change. Silages treated or untreated with a microbial inoculant, in three replicated bunker silos each, were used to create a robust data set to better examine patterns of T_si_ and pH change after exposure to air, the dual-sensor, and relationships with organic acid content. Comparison of the silage treatments per se was not the study purpose.

## Results

### Initial information on the two silages

According to a quality classification for maize silage^5^, initial counts of yeast and mold suggest excellent aerobic stability of the additive-treated silage, but reduced aerobic stability in the untreated silage (Table 1). In addition, the amount of acetic acid, and the ratio between lactic and acetic, in the additive-treated silage were not consistent with a maize silage that underwent heterolactic fermentation^1^.

**Table 1:** Chemical and microbial analyses of the two silages at sampling, not treated or treated with a biological additive.

	Control	Treated
Dry matter (g/kg WW)	421 ± 2.6	370 ± 4.1
pH	3.78 ± 0.07	3.62 ± 0.05
Buffering capacity (meq/100 g DM)	45.7 ± 0.56	48.4 ± 0.42
Lactic acid (g/kg DM)	51.5 ± 1.23	57.6 ± 0.79
Acetic acid (g/kg DM)	14.2 ± 0.43	16.0 ± 0.39
Butyric acid (g/kg DM)	< 0.3	< 0.3
Ethanol (g/kg DM)	6.24 ± 0.26	7.18 ± 0.25
Water soluble carbohydrate (g/kg DM)	21.4 ± 0.62	24.4 ± 0.55
Yeasts (log_10_ cfu/g WW)	5.21 ± 0.13	3.22 ± 0.15
Molds (log_10_ cfu/g WW)	< 2	< 2
Lactic acid bacteria (log_10_ cfu/g WW)	6.86 ± 0.23	7.11 ± 0.28
Total bacteria count (log_10_ cfu/g WW)	7.08 ± 0.23	7.27 ± 0.35

Data are means ± SE of n = 3 replicates; WW, wet weight; DM, dry matter; cfu, colony-forming units.

### Aerobic responses of T_si_ and pH

**Figure 1:**
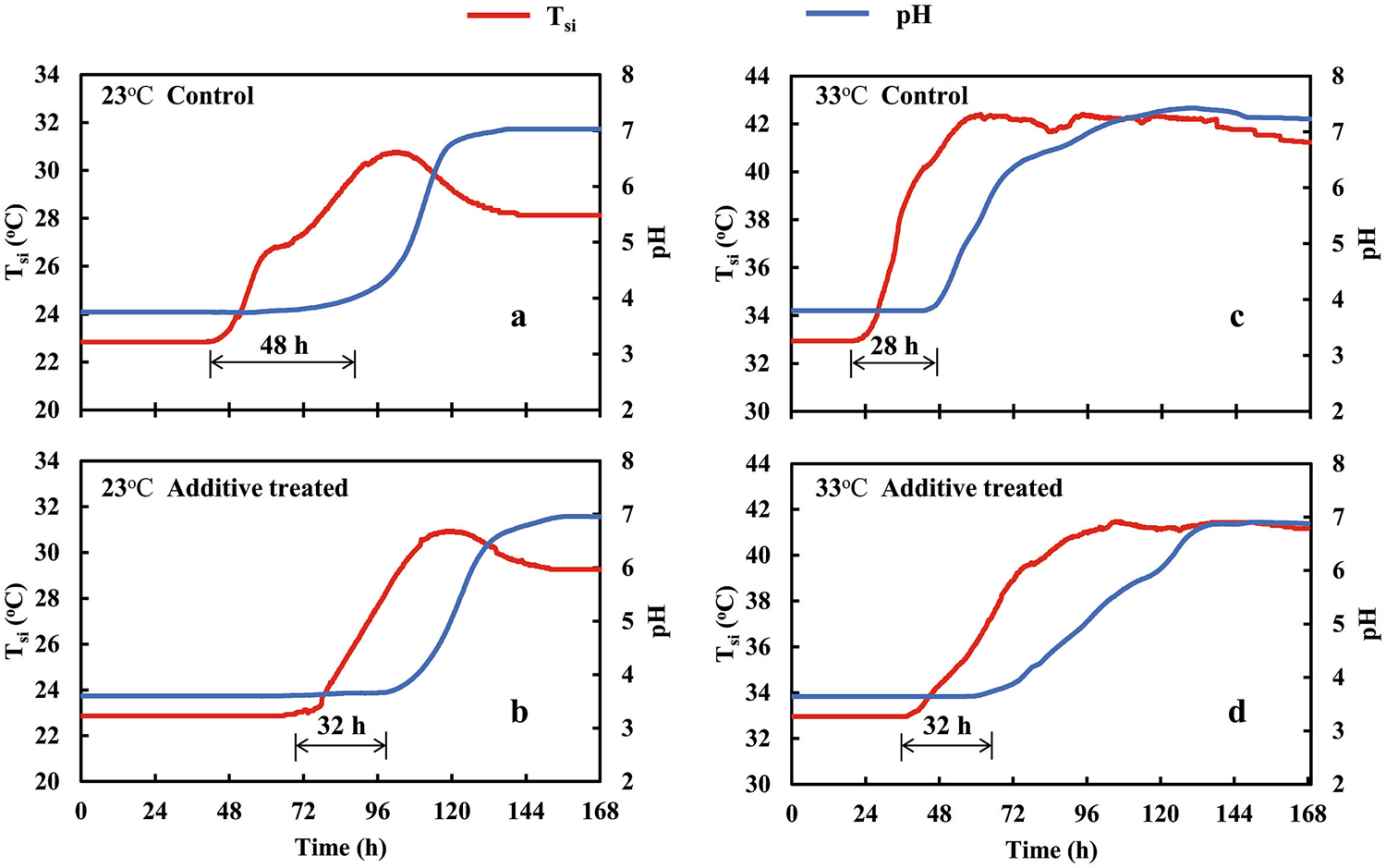
Silage temperature (T_si_) and pH patterns in control (**a**, **c**) or biological additive-treated (**b**, **d**) maize silage samples incubated at 23 °C (**a**, **b**) or 33 °C (**c**, **d**) for 168 h of incubation (T_c_).

The time courses of pH and T_si_ (each course represents the mean of three replicates) from the silages incubated at 23 °C (T_c_) (Fig. 1a,b) and 33 °C (Fig. 1c,d) were similar. Increases of T_si_ began earlier for 33 °C silage following exposure to air. Assuming that:

T_si_ – T_c_ = 2 °C is the threshold of instability^7–10^, the time sequence of these samples to cross the threshold was: control (30 h, 33 °C; 54 h, 23 °C) and treated (52 h, 33 °C; 84 h, 23 °C). This resulted in lower final lactic acid contents for 33 °C incubated silage than 23 °C (Table 2). The T_si_ at 23 °C increased with a subsequent decline, whereas T_si_ at 33 °C increased to plateau. Generally, there was a lag of 28 to 48 h between the onset of a T_si_ increase and onset of a pH increase following aerobic exposure.

**Table 2:** Final (*i.e*., 168 h of aerobic incubation) chemical and microbial analyses of the two silages, not treated or treated with a biological additive.

	Control				Treated			
	23 ℃	33 ℃	SEM	*P*	23 ℃	33 ℃	SEM	*P*
Dry matter (g/kg WW)	405	411	2.9	NS	359	358	2.3	NS
pH	7.0	7.2	0.098	NS	6.88	6.84	0.200	NS
Lactic acid (g/kg DM)	6.21	4.13	0.149	**	8.47	4.87	0.171	**
Relative loss of lactic acid (%)	87.9	92.0	0.41	**	85.3	91.6	0.31	**
Acetic acid (g/kg DM)	5.24	3.27	0.154	**	6.06	5.69	0.129	NS
Butyric acid (g/kg DM)	< 0.38	< 0.38	–	–	< 0.38	< 0.38	–	–
Water soluble carbohydrate (g/kg DM)	6.95	5.87	0.189	*	7.66	6.26	0.296	*
Ethanol (g/kg DM)	3.89	2.89	0.156	*	2.56	2.76	0.113	NS
Yeasts (log_10_ cfu/g WW)	5.83	7.79	0.290	**	6.52	5.52	0.360	NS
Molds (log_10_ cfu/g WW)	< 2	< 2	–	–	< 2	< 2	–	–
Lactic acid bacteria (log_10_ cfu/g WW)	7.14	7.33	0.448	NS	8.28	8.64	0.212	NS
Total bacteria count (log_10_ cfu/g WW)	7.74	7.42	0.449	NS	8.31	8.51	0.264	NS

WW, wet weight; DM, dry matter; cfu, colony-forming units.

*, *P* < 0.05; **, *P* < 0.01; NS, *P* > 0.05.

### Validation of the in situ measurement of pH

**Figure 2:**
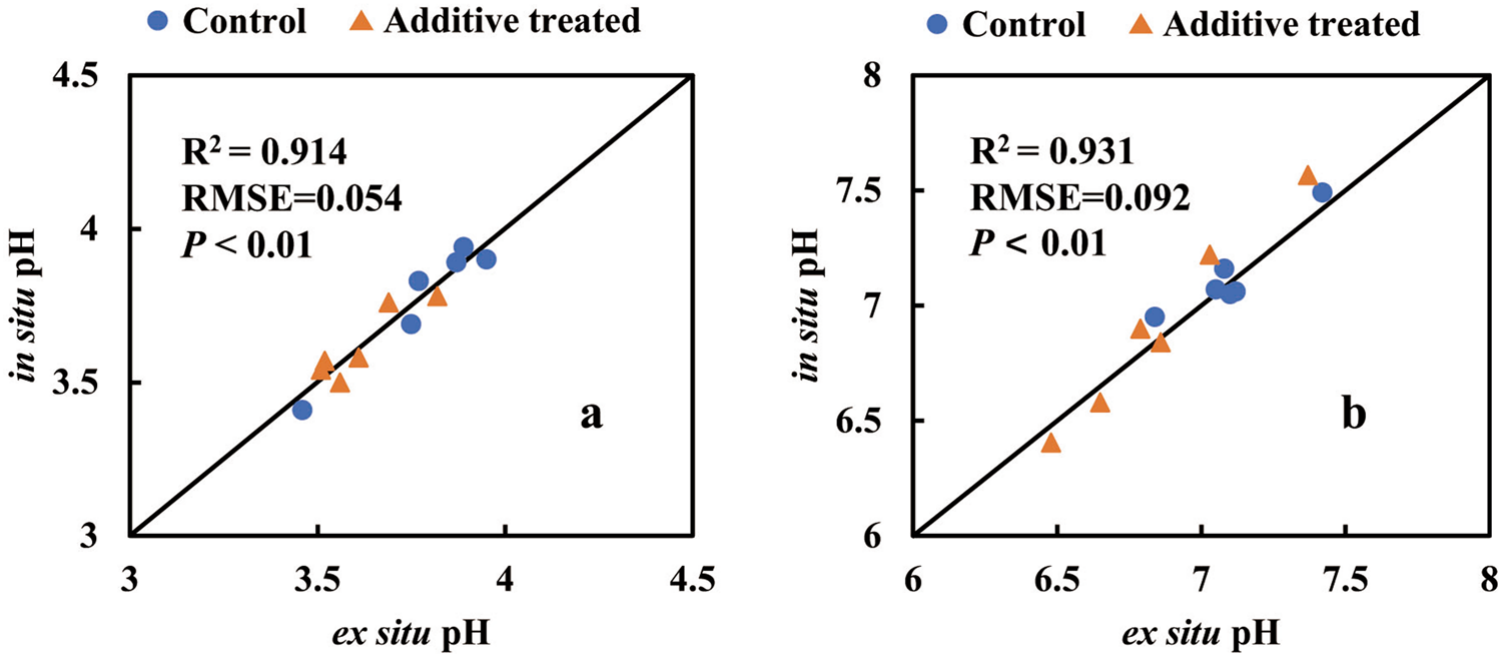
Piecewise comparison of in situ and ex situ pH in the 3.5 to 7.5 range (low pH group obtained before the experiment; high pH group at the end of aerobic exposure). The solid line is the line of equality. 24 paired data in the subfigures were measured from the samples in the same instrumented jar each.

Figure 2 (*n* = 12 × 2) shows the piecewise relationship between silage pH resulting from water-extraction (*i.e., *ex situ determination) and direct measurement (*i.e., *in situ), where paired values of in situ and ex situ were measured from the same instrumented jar/pH-sensor. The in situ and ex situ measurements agreed well, and were consistent with a 1:1 relationship of (*i.e.,* R^2^ = 0.914, RMSE = 0.054, *P* < 0.01) over the pH range of 3.5 to 4 (Fig. 2a, initial period) and (R^2^ = 0.931, RMSE = 0.092, and *P* < 0.01) over the pH range 6.5 to 7.5 (Fig. 2b, final period).

**Figure 3:**
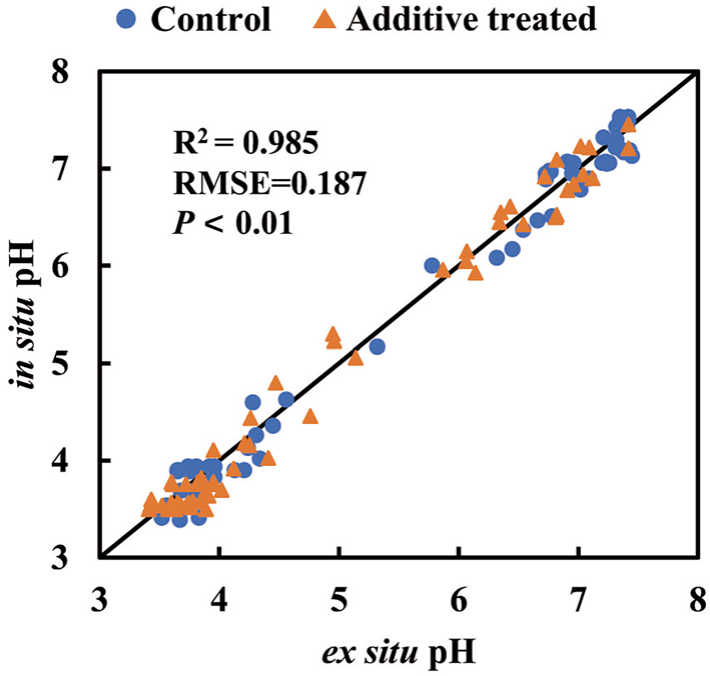
Comparison of in situ data from the 12 instrumented jars and ex situ data from the additional 120 jars. The solid line is the line of equality.

A general comparison between the additional jars with ex situ and in situ determinations using the instrumented jars over the process of experiment is in Fig. 3 (*n* = 120). In contrast to Fig. 2, the higher R^2^ of 0.985 and higher RMSE (0.187) is likely due to more data. Nevertheless, both Figs. 2 and 3 demonstrate that in situ pH measurements from 3.6 to 7.4 (Fig. 1) were accurate, and that the dual-sensor technique suited the mini-silos.

### Temporal variation of pH versus lactic and acetic acids

**Figure 4:**
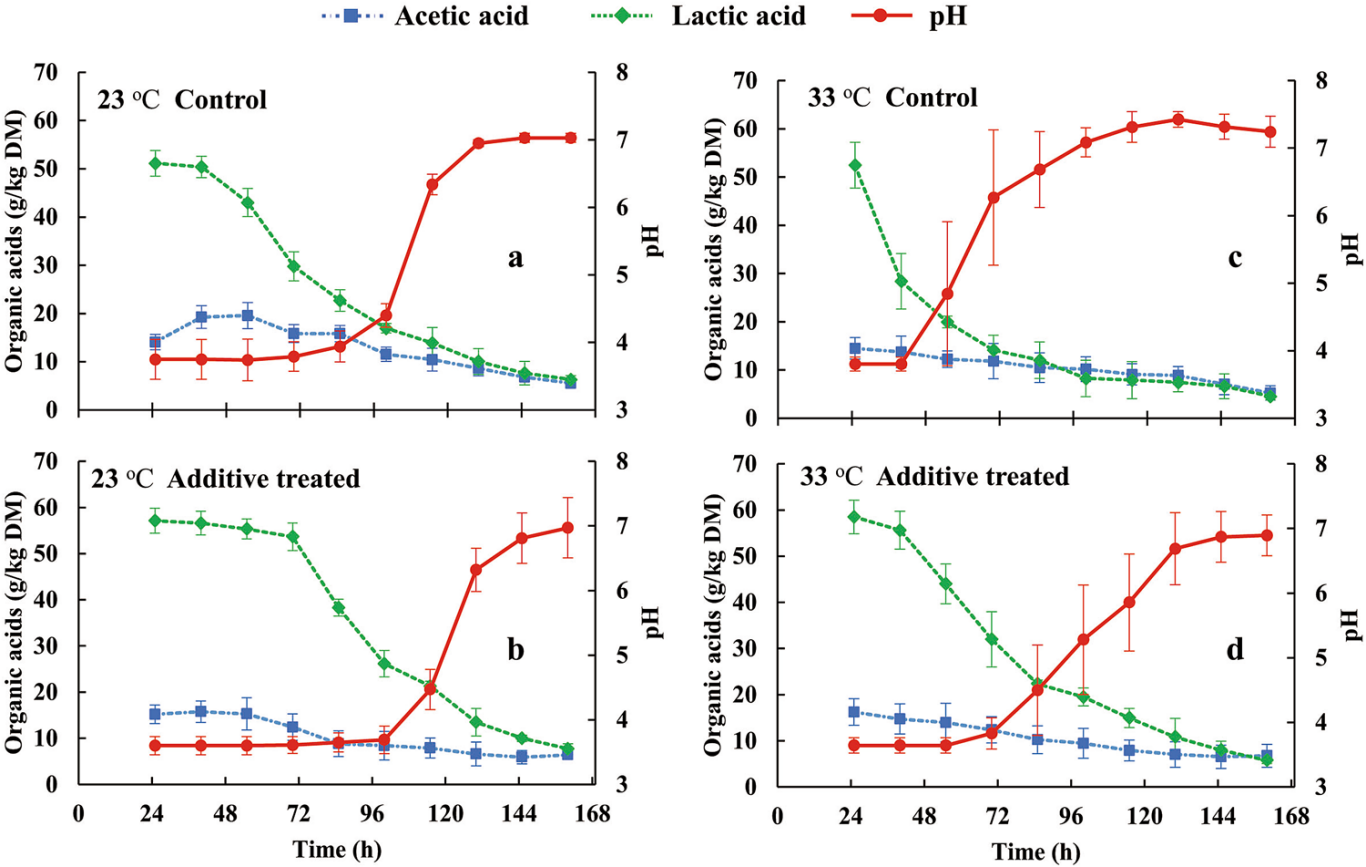
Acetic acid, lactic acid and pH patterns in control (**a**, **c**) and biological additive-treated samples (**b**, **d**) at 23 °C (**a**, **b**) or at 33 °C (**c**, **d**) for 168 h of incubation.

Profiles of pH *versus* lactic and acetic acids over time in silages incubated at 23 °C and 33 °C are present in Fig. 4. For those incubated at 23 °C, the lactic acid content began to decline at 40 h (control) or 70 h (treated silages) and continued to decline to very low levels. In contrast, lactic acids of these silages incubated at 33 °C decreased earlier and faster, reflecting the influence of the incubation temperature on microbial metabolic activity. The lactic acid contents in all samples were initially higher than those of acetic acid which, in the silages incubated at 23 °C, increased between 24 and 48 h, then declined to low levels. In general, the patterns in Fig. 4 show that most lactic acid in each sample was metabolized before the pH began to increase.

### Estimating relative loss of lactic acid (RLLA)

**Figure 5:**
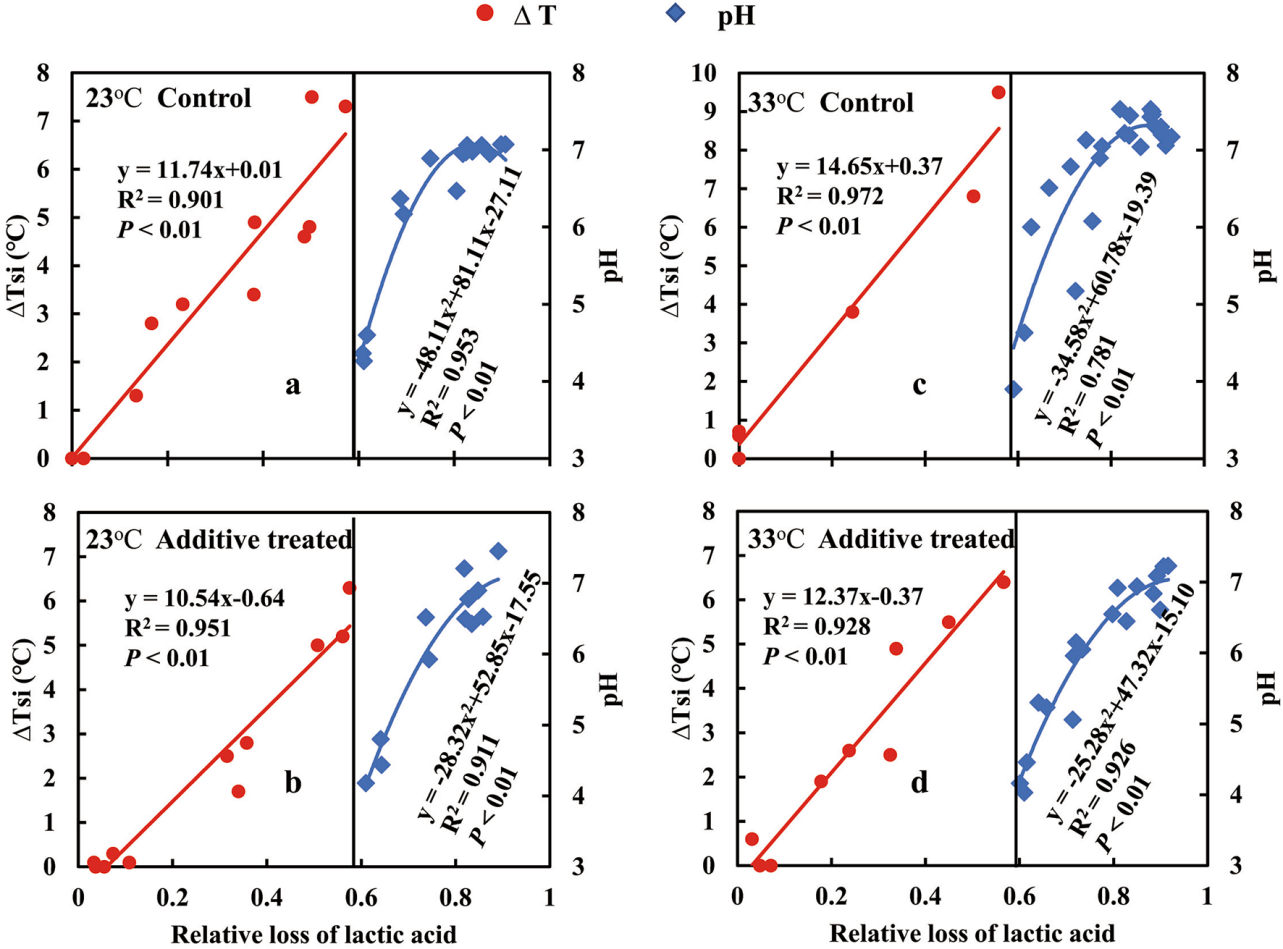
Estimates of the relative loss of lactic acid (RLLA) in the control silage (**a**, **c**) and biological additive-treated silage (**b**, **d**) at 23 °C (**a**, **b**) and at 33 °C (**c**, **d**) using T_si_ and pH.

Figures 1 and 4 suggest that the relative loss of lactic acid in these maize silages can be estimated from the combined information in the time courses of T_si_ and pH. In the initial 2 to 3 days, lactic acid declined but remained the primary organic acid (Fig. 4), although it is likely that buffering capacity of the silages mitigated a pH change despite the decline of lactic acid. This allowed RLLA to be estimated as: ΔT_si_ = T_si_ – T_c_ as an index of its loss (Fig. 5). The relationships of RLLA (0–60%) and ΔT_si_ (0 to 6–10 °C), on the left side of Fig. 5, were linear (R^2^ ≥ 0.901, *P* < 0.01). After T_si_ stabilized (Fig. 1), RLLA could be estimated from the quadratic increase in pH (R^2^ ≥ 0.781, *P* < 0.01; right side of Fig. 5). The non-linear relationship between pH and RLLA was likely due to changes in the acetic acid content. In the final stage, contents of lactic and acetic acids were similar (Fig. 4). Thus, for these maize silages over the range of 0 to 60% loss of lactic acid, ΔT_si_ was a useful predictive tool. However, as RLLA became ≥ 60%, estimation based on pH became more appropriate, albeit increasingly uncertain due to the non-linear relationship between pH and RLLA (right side of Fig. 5).

## Discussion

The slower changes in pH versus T_si_ over time of incubation after air exposure may reflect buffering capacity of the silage^4,10^. Since buffering is a normal property of plant material^26^, a time lag is inherent but likely situationally specific. During the lactic acid generating fermentation phase, a delayed pH decline has been observed^27–29^. However, effects of buffering on this lag time relative to the pH increase after silo opening have not been adequately addressed, although a potential role of buffering in aerobic stability was previously suggested based upon limited experimental support^1,30^. Our results, measured in situ, provide the first characterization of the time lag between T_si_ and pH in response to aerobic exposure, and demonstrate that a delayed pH increase during feed-out (*i.e.,* de-acidification) is affected by the concentration of lactic acid and buffering capacity of the silage mass.

In a study where maize silage was exposed to air^31^, the authors noted that yeast counts increased from 3 to 6.5 (log_10_ cfu/g) while pH remained < 4 in the initial 130 h. Thereafter, pH increased rapidly to 6.3 by 20 h. The authors^31^ attributed this outcome to buffering capacity, and presented numerous models associated temporal counts of yeast and mold to predict buffering capacity^26^. Tables 1 and 2, show that silage yeast counts increased at both incubation temperatures, and the only slight increase of acetic acid at 23 °C between 24 to 48 h (Fig. 4a) could be related to the microbial profile of yeasts over time.

pH has been regarded as an indicator/inhibitor of microbial activity/growth in silage^1,22^, with a lower pH reflecting stronger suppression to microbial activity^30,32^. However, simultaneous measurements of T_si_ and pH (Fig. 1) during the early unstable period of silage after exposure to air demonstrate the immediate onset of aerobic activity based on the T_si_ increase. That pH remained low for an additional 1 to 2 days is likely due to the buffering capacity of the maize silage. Overall, this suggests that pH may be an inadequate measure of microbial activity when silage is in the early aerobically unstable phase after silo opening^31^.

The pH time courses were dominated by variations in lactic acid during the initial period (Fig. 4) since its contents were higher than those of acetic acid. However, contents of these acids later converged (Fig. 4). Even with similar concentrations, it was likely lactic acid which was the primary pH determinants because the pKa of lactic acid (3.86), its acid dissociation constant, coincided with the lowest pH value (3.6, Fig. 1), whereas the pKa value of acetic acid is higher (4.75) reflecting it being a ten times weaker acid than lactic acid^30,33,34^. Thus, as the weaker acid, acetic had more undissociated molecules in silage water when the pH was < 4.75^34^. In addition, as acetic acid is more volatile than lactic^2,29,30^, and thus more acetic acid would have been lost by volatilization. Thus, pH is a useful indicator of aerobic loss of lactic acid (right sides of Fig. 5), but only after buffering capacity is exceeded.

Using this novel dual-measurement technique we found that pH is not as effective T_si_ as an earlier aerobic marker of maize silage spoilage, but is effective at longer times of air exposure. Indeed, both T_si_ and pH have been suggested as indicators of oxidative degradation^5,10,13,21,35–38^. The (ΔT_si_ =  + 2 °C) as the threshold of aerobic stability has been widely accepted^1,7,8^, while other studies have suggested a pH-based threshold of aerobic deterioration when pH exceeds the initial value by 0.5 units^17,39–41^. Our case study suggests that: ΔT_si_ =  + 2 °C, represents a 11 to 25% loss of lactic acid from these maize silages whereas: ΔT_si_ =  + 3 °C, also a commonly accepted threshold of aerobic deterioration, represents 18 to 35% loss. In contrast, at ΔpH = 0.5 unit, over 60% of lactic acid had been lost, thereby further supporting the suggestion^21^ that pH is a less sensitive indicator of aerobic deterioration than is T_si_ for maize silage. However, for farm silos where ambient temperature fluctuates diurnally^5,10,21^, this may interfere with determining the threshold of aerobic stability. As the chemical definition of pH is independent of ambient temperature, this may be an additional advantage for outdoor investigation.

Extrapolation of our outcomes with maize silage to grass and/or legume silages should be done with care as maize silage has a relatively low acid buffer capacities^42–44^ for a silage. Consequently, silage with higher acid buffer capacity may exhibit different pH time delays relative to T_si_ in response to air exposure and subsequent aerobic deterioration.

## Conclusions

Time courses of T_si_ and pH simultaneously measured in maize silages in situ using a novel dual-sensor documented lags of pH change relative to those in T_si_ at two incubation temperatures. These lags, likely due to ongoing losses of lactic acid and silage buffering capacity that delayed the pH rise following aerobic exposure, demonstrate that pH is a less sensitive indicator than T_si_ of short time aerobic deterioration of maize silage. However, pH is an effective indicator of advanced spoilage and loss of lactic acid due to aerobic metabolism.

Our results refer to a case observation using the dual-sensor tool that is different from ex situ method. We presented the comparison/discussion of the in situ and ex situ data and outcomes recommend this simpler and faster in situ method for mini-silo studies. As all maize samples measured were extracted non-destructively from farm silos, this technique is promising for farm level use, although a protective shield for the glass electrode of pH sensor is necessary. The stronger conclusion that the temperature outperforms pH as an early sign of aerobic loss of various silage depends on extending experiments, associated with multiple affecting factors in future study.

## Methods

### Experimental materials

A total of 132 glass jars (2 (two farms that provided different silage samples) × 3 (three silos of each farm for replicates) × 2 (incubated at 23 °C and 33 °C) × 11 (1 in situ + 10 ex situ measurements)) were filled with whole crop maize (*Zea mays* L.) silage. Control samples (66 jars, sampling date June 17, 2019) were collected from three bunker silos (40 × 6 × 3.5 m^3^) located at the Frankenforst Research Farm (University of Bonn, Bonn, Germany). Additive treated samples (66 jars, sampling date June 29, 2019) were collected from three bunker silos (30 × 5 × 2.5 m^3^) at a nearby private farm where these silos were inoculated at harvest with a biological additive containing *Lactobacillus buchneri*, *Lactobacillus plantarum*, and *Lactobacillus rhamnosus* (BONSILAGE FIT M (liquid), 170713, homo- and hetero-fermentative, H. Wilhelm Schaumann GmbH, Germany) at 1 g/t wet weight). Both farms cultivated cv. Susann S260 (Saaten Union, Germany) with similar seeding (late April, 2018) and the same harvest (August 28, 2018) dates, and had similar unloading rates (0.5 m/d).

**Figure 6:**
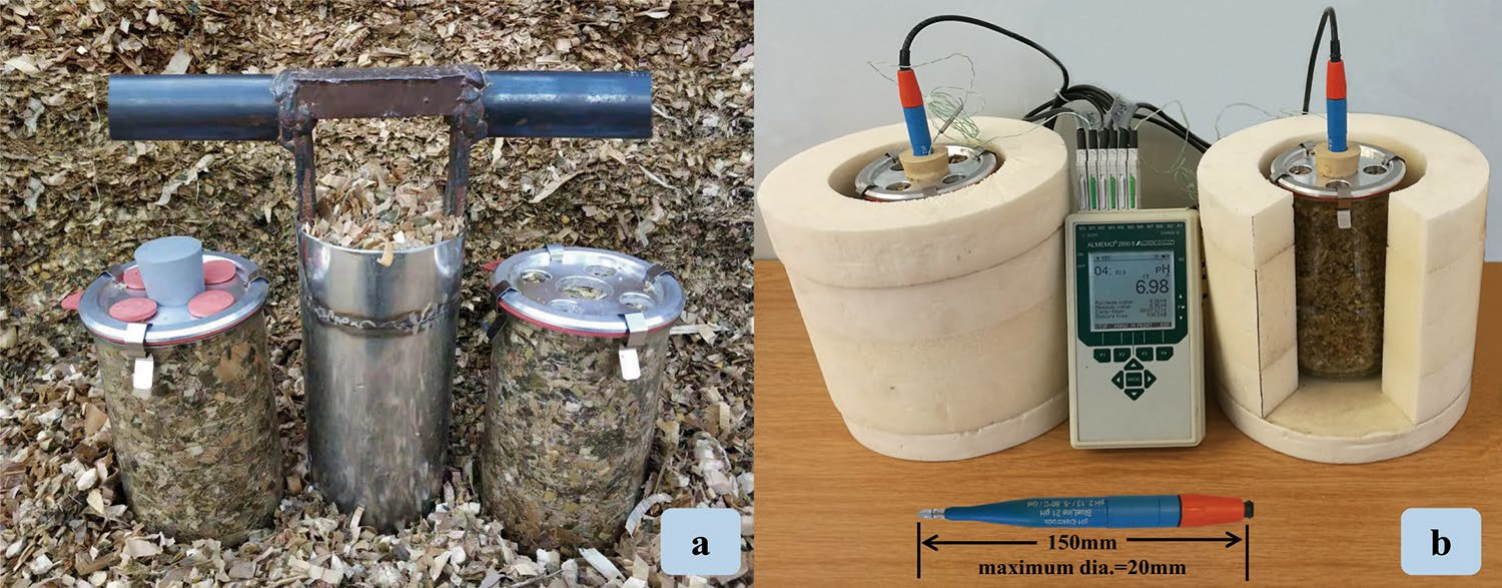
Silage probed using the core sampling device and immediately packed into jars in the on-farm silo (**a**), and the instrumented jars with insulating cover ready for laboratory incubation (**b**).

To facilitate sampling from the silos, while minimizing air entry, a metallic coring device (inner diam. 9.5 cm, length 25 cm; Fig. 6a) was constructed with the same inner diameter as the glass jars (1.5 L, inner diam. 10 cm, depth 20 cm; Fig. 6a) into which the samples were placed for deterioration assessment. The sampling area (1.8 m × 1.4 m) was located in the center of each silo. Prior to sampling from the bunker, 20 cm of the surface of the exposed face was removed. Sampled silage was immediately packed to a density of 220 kg/m^3^ dry matter (DM) in glass jars which were tightly sealed with aluminum lids (diam. 10 cm, thickness 1 cm), rubber O-rings and four elastic clamps (Fig. 6a).

### In situ silage pH sensor

The study required validation that the pH sensor used is suitable for liquids and maize silage with typically high water content (> 600 g/kg) under high compaction/density (500 to 600 kg/m^3^ wet weight). Since maize silage is a H_2_O-rich porous material^45^, its dense compaction enables good contact between the pH-electrode and the water phase in the silage. The lids of the glass jars were perforated with five holes (Fig. 6b), with the center hole (diam. 3 cm) allowing insertion of a glass pH electrode (2–13 pH/ ± 0.01 pH, BlueLine 21, SI Analytic GmbH, Mainz, Germany; Fig. 6b) and a thermocouple (NiCr-Ni, diam. 1 mm, − 40–160 °C/ ± 0.1 °C, Alborn Mess-und Regelungstechnik GmbH, Germany). The other holes (diam. 2 cm) were air inlets for the aerobic-exposure measures when rubber stoppers were removed. Ex situ determination of silage pH followed a standard protocol of extraction of 25 g of maize silage with 0.225 L deionized water for 30 min^4,14,23^.

### Experimental procedure

The experiment used temperatures of 23 and 33 °C (T_c_) in an incubator (KBF-S 720, control range: 0 to 70 °C, accuracy: ± 0.3 °C, vol. 0.97 × 0.58 × 1.25 m, BINDER GmbH, Tuttlingen, Germany). According to a suggestion^36^, all mini-silos/jars were covered with insulated sleeves throughout (Fig. 6b). Prior to and after the experiment, all pH sensors of the instrumented jars were calibrated with standard calibration liquids: HI 70,004 (pH = 4.01) and HI 70,007 (pH = 7.01), (Hanna Instruments, Inc. Woonsocket, USA). The pH tip and a thermocouple (1 mm diameter, T-type) were inserted together into the sample at the same depths (8 cm) in each jar. Both sensors are connected to a data-logger (ALMEMO-2890–9, AHLBORN GmbH, Germany) with data recorded at 10 min intervals over the 168 h experimental cycles. Each instrumented jar, and 10 additional jars (1.5 L) as a group all containing the same maize silage and packed to the same density. These additional jars served as sampling points during aerobic deterioration measures. During each sampling (15 h interval beginning 24 h after ensilage), a subsample of 150 g was removed from 8 cm behind the face in these additional jars, to same depth as the in situ pH and thermocouple sensors. A 100 g subsample was sealed with a vacuumizer (Boss Mini-Max, Helmut Boss Verpackungsmaschinen KG, Bad Homburg, Germany) to remove air, frozen and shipped on ice to a commercial laboratory for chemical and microbial analyses within 24 h. The remaining 50 g subsample was divided equally, each with 225 g deionized water, to determine pH ex situ, which used for evaluation of in situ the measurements from the instrumented jars.

### Statistical analysis

Experimental data were analyzed using IBM SPSS v25.0 (IBM Co., Armonk, NY, USA). Linear regression, curve fitting and fitting errors were evaluated using coefficient of determination (R^2^), significance (P) and root mean square error (RMSE). A T-test was conducted to determine effect of T_c_ (i.e., incubated at 23 or 33 °C) on chemical and microbial composition at the end of the experiment for final-data processing.

### Chemical analysis

Dry matter was measured by drying at 60 °C for 48 h in a forced-air oven^39^. Buffering capacity (BC) was determined by the method of the literature^46^. Acids (*i.e.,* lactic, acetic, butyric) and ethanol were determined using high-performance liquid chromatography (LC-2010AHT, Shimadzu Corp., Kyoto, Japan), with an integrated UV-detector. Water soluble carbohydrate (WSC) content was determined enzymatically^47^.

### Microbial analysis

According to the method of the literature^3^, 30 g of silage was suspended in 270 ml of ¼-strength ringer solution (2.25 g/ l NaCl, 0.105 g/l KCl, 0.06 g/l CaCl2, 0.05 g/l NaHCO3) (Merck, Darmstadt, Germany) and homogenized in a mixer for one minute. From this suspension, total bacterial counts were analyzed on plate-count agar (5.0 g/l enzymatic digest of casein, 2.5 g/l yeast extract, 1.0 g/l glucose, 15 g/l agar, pH = 7.0) (Merck, Darmstadt, Germany) after aerobic incubation at 30 °C for 2 days. Lactic acid bacteria were quantified on MRS agar (Merck, Darmstadt, Germany) after anaerobic cultivation for 3 days at 30 °C. Yeasts and molds were detected using yeast extract glucose chloramphenicol (YGC)-agar (5.0 g/l yeast extract, 20.0 g/l glucose, 0.1 g/l chloramphenicol, 14.9 g/l agar, pH = 6.6) (Merck, Darmstadt, Germany) after incubation at 25 °C for 3 days.

## References

[1] Wilkinson JM, Davies DR (2013). The aerobic stability of silage: key findings and recent developments. Grass Forage Sci..

[2] Weiss K, Kroschewski B, Auerbach H (2016). Effects of air exposure, temperature and additives on fermentation characteristics, yeast count, aerobic stability and volatile organic compounds in corn silage. J. Dairy Sci..

[3] Li M (2017). CO_2_ production, dissolution and pressure dynamics during silage production: multi-sensor-based insight into parameter interactions. Sci. Rep..

[4] Woodard KR, Prine GM, Bates DB (1991). Silage characteristics of elephantgrass as affected by harvest frequency and genotype. Agron. J..

[5] Robinson PH, Swanepoel N (2016). Impacts of a polyethylene silage pile underlay plastic with or without enhanced oxygen barrier (EOB) characteristics on preservation of whole crop maize silage, as well as a short investigation of peripheral deterioration on exposed silage faces. Anim. Feed Sci. Technol..

[6] Johnson LM (2002). Corn silage management: effects of maturity, inoculation, and mechanical processing on pack density and aerobic stability. J. Dairy Sci..

[7] Ranjit NK, Kung L (2000). The effect of *Lactobacillus buchneri*, *Lactobacillus plantarum*, or a chemical preservative on the fermentation and aerobic stability of corn silage. J. Dairy Sci..

[8] Muck RE (2004). Effects of corn silage inoculants on aerobic stability. Trans. ASAE.

[9] Puntillo M (2020). Potential of lactic acid bacteria isolated from different forages as silage inoculants for improving fermentation quality and aerobic stability. Front. Microbiol..

[10] Borreani G, Tabacco E (2010). The relationship of silage temperature with the microbiological status of the face of corn silage bunkers. J. Dairy Sci..

[11] Kristensen NB (2010). Effects of microbial inoculants on corn silage fermentation, microbial contents, aerobic stability, and milk production under field conditions. J. Dairy Sci..

[12] Jatkauskas J, Vrotniakiene V, Ohlsson C, Lund B (2013). The effects of three silage inoculants on aerobic stability in grass, clover-grass, lucerne and maize silages. Agric. Food Sci..

[13] Herrmann C, Idler C, Heiermann M (2015). Improving aerobic stability and biogas production of maize silage using silage additives. Bioresour. Technol..

[14] Liu B (2020). Impact of molasses and microbial inoculants on fermentation quality, aerobic stability, and bacterial and fungal microbiomes of barley silage. Sci. Rep..

[15] O’Kiely P, Muck RE (1992). Aerobic deterioration of lucerne (*medicago sativa*) and maize (*zea mais*) silages-effects of yeasts. J. Sci. Food Agric..

[16] Ashbell G, Lisker N (1988). Aerobic deterioration in maize silage stored in a bunker silo under farm conditions in a subtropical climate. J. Sci. Food Agric..

[17] Liu Q, Zhang J, Shi S, Sun Q (2011). The effects of wilting and storage temperatures on the fermentation quality and aerobic stability of stylo silage. Anim. Sci. J..

[18] Yuan X (2015). The effect of different additives on the fermentation quality, in vitro digestibility and aerobic stability of a total mixed ration silage. Anim. Feed Sci. Technol..

[19] Addah W, Baah J, Groenewegen P, Okine EK, McAllister TA (2011). Comparison of the fermentation characteristics, aerobic stability and nutritive value of barley and corn silages ensiled with or without a mixed bacterial inoculant. Can. J. Anim. Sci..

[20] Tabacco E, Piano S, Revello-Chion A, Borreani G (2011). Effect of *Lactobacillus buchneri* LN4637 and *Lactobacillus buchneri* LN40177 on the aerobic stability, fermentation products, and microbial populations of corn silage under farm conditions. J. Dairy Sci..

[21] Okatsu Y, Swanepoel N, Maga EA, Robinson PH (2019). Impacts of some factors that effect spoilage of silage at the periphery of the exposed face of corn silage piles. Anim. Feed Sci. Technol..

[22] Borreani G, Tabacco E, Schmidt RJ, Holmes BJ, Muck RE (2018). Silage review: factors affecting dry matter and quality losses in silages. J. Dairy Sci..

[23] Junges D, Schmidt P, Novinski CO, Daniel JLP (2013). Additive containing homo and heterolactic bacteria on the fermentation quality of maize silage. Acta Sci. Anim. Sci..

[24] Wilkinson JM, Muck RE (2019). Ensiling in 2050: some challenges and opportunities. Grass Forage Sci..

[25] Bolsen KK, Ashbell G, Weinberg ZG (1996). Silage fermentation and silage additives—review. Asian-Australas. J. Anim. Sci..

[26] Courtin MG, Spoelstra SF (1990). A simulation model of the microbiological and chemical changes accompanying the initial stage of aerobic deterioration of silage. Grass Forage Sci..

[27] Pahlow, G., Muck, R. E., Driehuis, F., Elferink, S. J. O. & Spoelstra. S. F. Microbiology of ensiling in Silage Science and Technology. (ed. Buxton, D. R., Muck, R. E., & Harrison, J. H.) 31–93 (Madison, WI, 2003).

[28] Piltz, J. W. & Kaiser, A. G. Principles of silage preservation in Top Fodder Successful Silage. (ed. Kaiser, A. G., Piltz, J. W., Burns, H .M. & Griffiths, N. W.) 25–56 (NSW Australia, 2004).

[29] Kung L (2018). An evaluation of the effectiveness of a chemical additive based on sodium benzoate, potassium sorbate, and sodium nitrite on the fermentation and aerobic stability of corn silage. J. Dairy Sci..

[30] Kung L, Shaver RD, Grant RJ, Schmidt RJ (2018). Silage review: Interpretation of chemical, microbial, and organoleptic components of silages. J. Dairy Sci..

[31] Spoelstra SF, Couhtim MG, Van Beers JAC (1988). Acetic acid bacteria can initiate aerobic deterioration of maize silage. J. Agric. Sci..

[32] Holzer M, Mayrhuber E, Danner H, Braun R (2003). The role of *Lactobacillus buchneri* in forage preservation. Trends Biotechnol..

[33] Danner H, Holzer M, Mayrhuber E, Braun R (2003). Acetic acid increases stability of silage under aerobic conditions. Appl. Environ. Microbiol..

[34] Graves T, Narendranath NV, Dawson K, Power R (2006). Effect of pH and lactic or acetic acid on ethanol productivity by Saccharomyces cerevisiae in corn mash. J. Ind. Microbiol. Biotechnol..

[35] Henderson AR, Ewart JM, Robertson GM (1979). Studies on the aerobic stability of commercial silages. J. Sci. Food Agric..

[36] Honig, H. Evaluation of aerobic stability in Grass and forage reports. *Proceedings of the Eurobac Conference*, 76–82 (Uppsala, Sweden, 1990).

[37] Filya I (2004). Nutritive value and aerobic stability of whole crop maize silage harvested at four stages of maturity. Anim. Feed Sci. Tech..

[38] Ashbell G, Weinberg ZG, Hen Y, Filya I (2002). The effects of temperature on the aerobic stability of wheat and corn silages. J. Ind. Microbiol. Biotechnol..

[39] Weinberg ZG, Chen Y (2013). Effects of storage period on the composition of whole crop wheat and corn silages. Anim. Feed Sci. Technol..

[40] Chen L (2017). Effect of lactic acid bacteria and propionic acid on conservation characteristics, aerobic stability and in vitro gas production kinetics and digestibility of whole-crop corn based total mixed ration silage. J. Integr. Agric..

[41] Liu QH, Dong ZH, Shao T (2018). Effect of additives on fatty acid profile of high moisture alfalfa silage during ensiling and after exposure to air. Anim. Feed Sci. Technol..

[42] Davies DR (1998). Proteolysis during ensilage of forages varying in soluble sugar content. J. Dairy Sci..

[43] Meeske R, Basson HM (1998). The effect of a lactic acid bacterial inoculant on maize silage. Anim. Feed Sci. Tech..

[44] Buxton, D. R., & O’Kiely. P. Preharvest plant factors affecting ensiling in Silage Science and Technology. (ed. Buxton, D. R., Muck, R. E., & Harrison, J. H.) 199–250 (Madison, WI, 2003).

[45] Muck RE, Pitt RE (1994). Aerobic deterioration in corn silage relative to the silo face. Trans. ASAE.

[46] Playne MJ, McDonald P (1966). The buffering constituents of herbage and of silage. J. Sci. Food Agric..

[47] Madrid J, Martínez-Teruel A, Hernández F, Megías MD (1999). A comparative study on the determination of lactic acid in silage juice by colorimetric, high-performance liquid chromatography and enzymatic methods. J. Sci. Food Agric..

